# Factors Associated With Leaving-Without-Being-Seen in Pediatric Emergency Department Patients

**DOI:** 10.7759/cureus.75277

**Published:** 2024-12-07

**Authors:** Takashi Nihira, Takateru Ihara, Yuta Sasaoka, Yusuke Hagiwara

**Affiliations:** 1 Department of Pediatric Emergency Care and Intensive Care Medicine, Tokyo Metropolitan Children’s Medical Center, Tokyo, JPN; 2 Department of Emergency Medicine, Shonan Kamakura General Hospital, Kamakura, JPN; 3 Department of Pediatrics, Hyogo Prefectural Amagasaki General Medical Center, Amagasaki, JPN; 4 Department of Pediatrics, Hakodate Municipal Hospital, Hokkaido, JPN; 5 Department of Pediatric Emergency Care and Intensive Care Medicine, Tokyo Metropolitan Children's Medical Center, Tokyo, JPN

**Keywords:** acuity, emergency services, leaving-without-being-seen, triage, waiting time

## Abstract

Aim

Preventing leaving-without-being-seen (LWBS) in children is crucial due to their inability to seek medical care independently. Because there are no studies of LWBS in Japan, the extent of this problem in Japan and its impacts on healthcare are uncertain. The present study seeks to fill this gap by investigating LWBS after triage and identifying the associated factors.

Methods

The present, retrospective cohort study was conducted using an electronic, administrative database at a tertiary pediatric medical center in Japan. All records of children aged 15 years or less presenting to the emergency department between April 1, 2014 and March 31, 2017 were included, and the factors associated with LWBS were analyzed.

Results

During the study period, 112,059 patients were registered, of whom 168 (0.15%) were identified as having LWBS. Several factors were associated with LWBS, including less urgent acuity (odds ratio [OR]: 2.33) and visits on weekends/holidays (OR: 1.71) and evenings (OR: 1.44). Increased emergency department length of stay (EDLOS) and wait time were also associated with increased LWBS (OR: 3.48 for EDLOS > one hour; OR: 41.93 for waiting time > one hour).

Conclusion

Low acuity; visits on weekends/holidays, and evenings; EDLOS of more than one hour; and waiting time of more than one hour were associated with LWBS. These findings were in line with those of previous studies conducted in other countries, suggesting that they might be highly generalizable.

## Introduction

In the emergency department (ED), non-urgent patients are often forced to endure a prolonged wait, prompting some to leave the ED after triage, i.e., leaving without being seen (LWBS). LWBS poses various risks, including 1) exacerbation of a complaint due to delayed treatment; 2) reduced patient satisfaction; and 3) economic losses from triage without subsequent care [1]. Therefore, LWBS is a major concern for the ED and serves as an important, international, clinical quality index [2].

The Department of Health of the United Kingdom, in conjunction with the College of Emergency Medicine, had previously stated that an LWBS rate <5% was desirable for avoiding the risk of adverse events [3]. The LWBS rate varies widely among countries and institutions, ranging from 0.5-16.6% in pediatric emergency departments in children's hospitals [4-15] and 5.4-6.1% for pediatric emergencies in other emergency departments [16]. Previous reports of adult patients suggested that LWBS is associated with low-acuity triage, younger age, emergency department crowding, time of visit, emergency department length of stay (EDLOS), and post-triage waiting time [1,15-20]. EDLOS was also reportedly associated with boarding time. Although LWBS patients generally have a non-urgent complaint, some may urgently require attention. Some 1.3-7.3% of LWBS patients reportedly required hospitalization [6,14,18,21], while 50-78% later sought further medical care [5,6,21]. Preventing LWBS in children is critical, given their inability, unlike adults, to seek medical care independently. LWBS can hinder their access to suitable treatment. However, extrapolating from the currently available data from various hospitals worldwide is challenging because hospital practices, etc. vary internationally. For the results to be highly generalizable, the findings of a study must be applicable to healthcare systems across various regions.

As there are currently no studies of LWBS in Japan, the present study seeks to address this gap by examining LWBS after triage at the study center with the final aim of identifying the factors associated with LWBS.

## Materials and methods

The present, retrospective, cohort study used electronic, administrative data from a tertiary pediatric hospital in Tokyo and focused on the Pediatric Emergency Department (PED). This hospital has 561 beds, and the PED at the study center serves children aged 15 years or less and has an annual intake of around 37,000 patients (10% arriving by ambulance). Trauma patients accounted for 20% of all the PED visits. The hospital also functions as a teaching hospital and is supervised by board-certified pediatricians and senior emergency physicians.

Records of children aged 15 years or less who were seen and received triage in the PED between April 1, 2014 and March 31, 2017 were analyzed. Cancellations after triage were routinely recorded. The administrative staff systematically extracted the data from electronic medical records, and the corresponding author, T.N., reviewed the records of the patients with LWBS to determine their reason for leaving. Reasons for cancellations are obtained through interviews conducted by nurses and are subsequently recorded in the patients' medical records. Standardized forms are not used for this documentation. In cases where multiple reasons were given, both were counted. Specific variables in the study were predefined with reference to previous studies before the data were abstracted. Both the staff and the authors ensured that the data were accurately extracted. Blinding and testing for inter-rater agreement were omitted. The exclusion criteria were 1) insufficient documentation, such as the missing commencement time of consultations and 2) judgment by the principal investigator that a patient was unsuitable for enrollment. The following information was collected from the electronic database: age, sex, chief complaint, method of visit (walk-in or via ambulance), referral to the PED, visit date, visit time (8:00-15:59, 16:00-23:59, 0:00-7:59), day of the week (weekend, holiday, weekday), Japanese Triage and Acuity Scale for children (JTAS) triage level, EDLOS, waiting time, boarding time, and reason for leaving. In addition, the number of registered patients per day, time of day (8:00-15:59, 16:00-23:59, 0:00-7:59), and hour were investigated as factors in the PED that might have influenced the above items.

**Figure 1:**
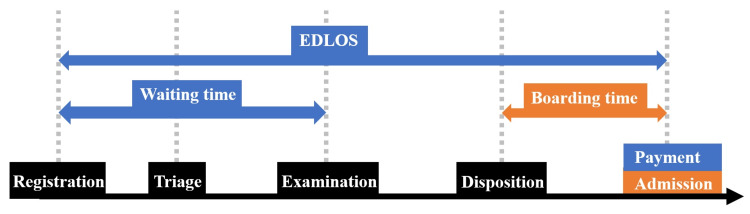
Definition of the time categories. In the LWBS group, waiting time was identical with EDLOS. EDLOS, emergency department length of stay; LWBS, leaving-without-being-seen.

Figure 1 provides the time category definitions [7]. EDLOS was defined as the time from registration to payment for the non-LWBS group. This is because the last recorded time in the electronic medical record was the payment time. For the LWBS group, EDLOS was defined as the time from registration to the request for discharge. Waiting time was defined as the time from registration to the start of the examination. Since no examination was conducted for the LWBS group, EDLOS and waiting time were, as a result, identical [8]. Payment in the non-LWBS group and leaving in the LWBS group were equivalent to departure. Waiting time, or the interval from registration to examination commencement, mirrored EDLOS for the LWBS group. The system automatically records registration and payment times in the electronic medical record upon task completion. The consultation start time is automatically logged when the physician initiates the medical record. Prognosis (length of stay or death) and adverse events (revisits within 72 hours, intravenous infusion, muscle injection, fracture reduction, fracture fixation, emergency surgery, hospitalization) were investigated [5,10,17,18,21,22].

Triage was performed by a registered nurse using the JTAS, a modified version of the Canadian Triage and Acuity Scale (CTAS). Under this system, patients are divided into five categories, each with an objective response time: JTAS 1 (resuscitation, immediate); JTAS 2 (emergent, 15 minutes); JTAS 3 (urgent, 30 minutes); JTAS 4 (less urgent, 60 minutes); and JTAS 5 (non-urgent, 120 minutes). The pediatric reference ranges for respiratory rate (RR) and heart rate (HR) recommended in the 2013 revision of the CTAS were used.

The primary outcome was the LWBS rate. The secondary outcome was factors found to be associated with LWBS by comparing the LWBS group with the non-LWBS group, for which boarding time was analyzed.

Tokyo Metropolitan Children’s Medical Center's institutional review board granted approval number 2019b-101 for this study, and all the analyses were conducted using Microsoft 365 Excel (Redmond, WA, USA). Significance was determined by the 95% confidence interval (CI).

## Results

**Figure 2:**
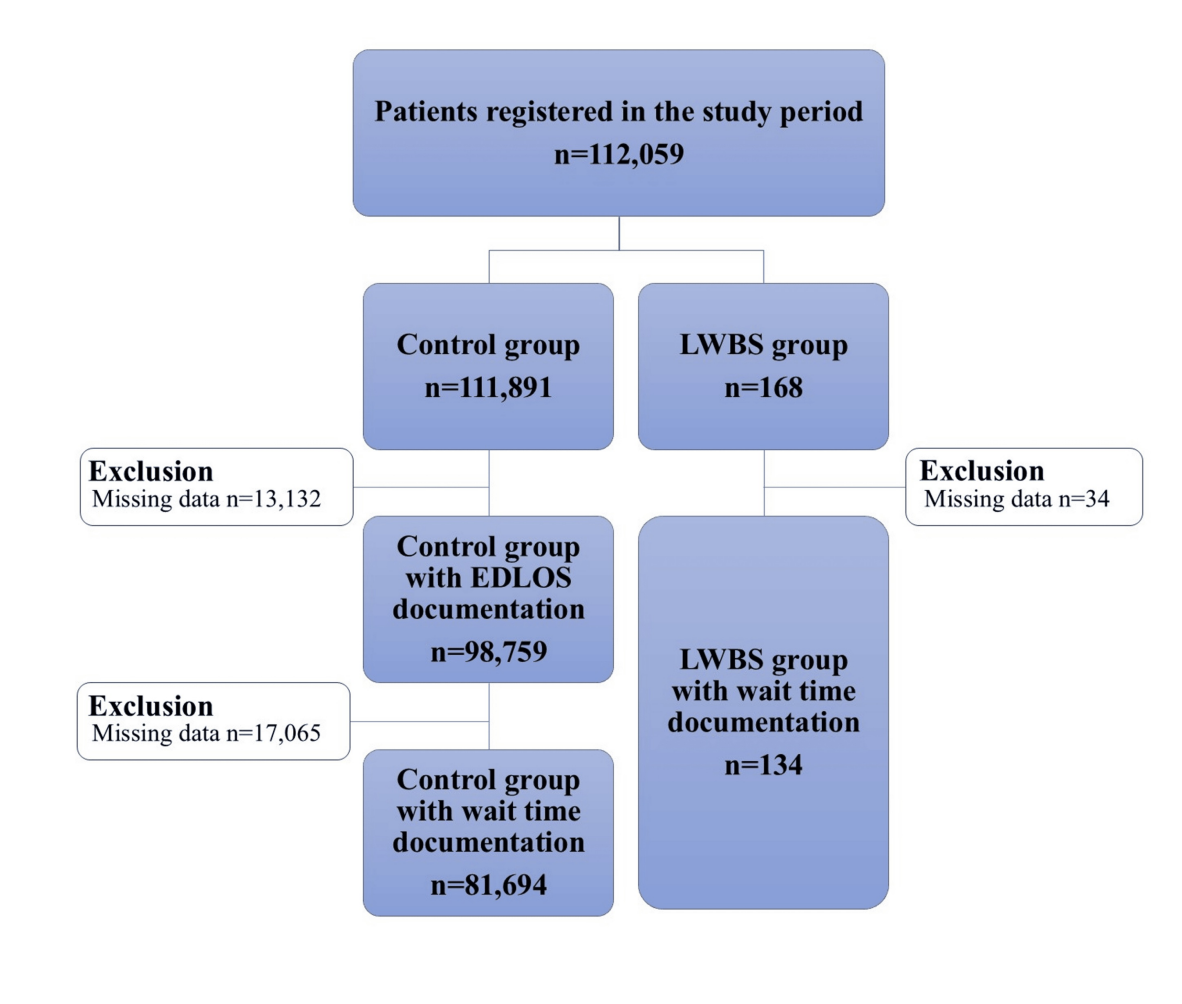
Patient flow diagram. EDLOS, emergency department length of stay; LWBS, leaving-without-being-seen.

Figure 2 illustrates the patient flow through the study. During the study period, 112,059 patients were registered in the PED, and 168 were identified as having LWBS. The median, annual LWBS rate was 0.15% (range: 0.08-0.17). No patients were judged unsuitable for participation by the investigator. Twelve patients (7.1%) with LWBS returned to the PED, and three of these had a higher triage category than at the initial visit. Only one patient (0.6%) received an intravenous infusion followed by hospitalization. None of the remaining 11 patients required medical intervention or hospitalization. There were no deaths in the LWBS group.

LWBS occurred at triage levels below “urgent.” Compared with the non-LWBS group, “less urgent” had the largest odds ratio (OR) but the “non-urgent” did not show statistical significance (urgent, OR: 0.66; 95% CI: 0.48-0.91; less urgent, OR: 2.33; 95% CI: 1.71-3.19; non-urgent, OR: 1.41; 95% CI: 0.52-3.80). There was also no significant difference in age. Some 58.9% of LWBS cases occurred on weekends/holidays (OR: 1.71; 95% CI: 1.26-2.33), and 57.7% occurred in the evening (16:00-23:59) (OR: 1.44; 95% CI: 1.06-1.96) (Table 1).

**Table 1:** Comparison of the clinical characteristics of LWBS group and control group. CI, confidence interval; PED, pediatric emergency department; JTAS, Japanese Triage and Acuity Scale; EDLOS, emergency department length of stay; LWBS, left without being seen; OR, odds ratio.

Characteristics	LWBS		Control		OR	95%CI		
	(n-168)		(n=111,891)					
Sex								
Male	86	51%	64,246	57%	0.78	0.58	-	1.05
Mode of arrival								
Ambulance	3	2%	9,443	8%	0.20	0.06	-	0.62
Helicopter	0		7	0%				
Referral to the PED								
Referred	3	2%	7,554	7%	0.25	0.08	-	0.79
JTAS category								
1 (resuscitation)	0	0%	1,415	1%				
2 (emergent)	0	0%	11,687	10%				
3 (urgent)	57	34%	48,868	44%	0.66	0.48	-	0.91
4 (less urgent)	107	64%	48,012	43%	2.33	1.71	-	3.19
5 (non-urgent)	4	2%	1,905	2%	1.41	0.52	-	3.80
not described	0	0%	4	0%				
Age								
< 3 months	2	1%	3,209	3%	0.41	0.10	-	1.65
3 to 11 months	12	7%	12,168	11%	0.63	0.35	-	1.13
1-2 years old	58	35%	31,725	28%	1.33	0.97	-	1.83
3-4 years old	37	22%	21,891	20%	1.16	0.81	-	1.67
5-11 years old	45	27%	34,028	30%	0.84	0.60	-	1.18
> 11 years old	14	8%	8,870	8%	1.06	0.61	-	1.83
Day of the week								
Weekends/holidays	99	59%	51,020	46%	1.71	1.26	-	2.33
Time period								
8:00-15:59	62	37%	44,415	40%	0.89	0.65	-	1.22
16:00-23:59	97	58%	54,405	49%	1.44	1.06	-	1.96
0:00-7:59	9	5%	13,071	12%	0.43	0.22	-	0.84

Increased EDLOS was associated with increased LWBS; the OR was 3.48 (95% CI: 2.54-4.76) for LWBS with EDLOS of more than one hour and even greater at 5.43 (95% CI: 4.01-7.37) for EDLOS of more than two hours. The strength of the association was even more remarkable when the focus was placed on waiting time as a component of EDLOS; the OR was 41.93 (95% CI: 30.57-57.51) for LWBS with waiting time of more than one hour and 359.56 (95% CI: 259.30-498.58) for waiting time of more than two hours. The proportion of patients with EDLOS within four hours was 97.2% in the non-LWBS group (Table 2).

**Table 2:** The comparison of LWBS group and control group about EDLOS and wait time after triage. CI, confidence interval; EDLOS, emergency department length of stay; LWBS, left without being seen; OR, odds ratio.

	LWBS	Control	OR	95%CI		
	(n=134)	(n=98,579)				
EDLOS						
Median	2:25	1:05				
1:00:00~	109	54,853	3.48	2.54	-	4.76
2:00:00~	73	17,790	5.43	4.01	-	7.37
	LWBS	Control	OR	95%CI		
	(n=134)	(n=81,694)				
Wait time after triage						
Median	2:25	0:24				
1:00:00~	109	7,695	41.93	30.57	-	57.51
2:00:00~	73	271	359.56	259.30	-	498.58

During the study period, 8487 patients were admitted; of these, 47 were excluded owing to missing or incomplete data, leaving 8,440 patients for the analysis. The average boarding time was 35 minutes. There was no difference in terms of the day of the week or time of the visit (Table 3).

**Table 3:** Average boarding time in emergency department.

	Boarding time (hr: min)	Hospitalization (n)
Day of the week		
Total	0:35	8440
Monday	0:37	1108
Tuesday	0:36	1075
Wednesday	0:37	1042
Thursday	0:38	1011
Friday	0:37	1192
Weekend and national holiday	0:31	3012
Time period		
8:00-15:59	0:41	3818
16:00-23:59	0:28	3614
0:00-7:59	0:35	1008

One patient left the hospital without notification. All the other LWBS patients had informed the nurse of their intention to leave. The reason for leaving was documented in 122 patients (Table 4). The most common reason given was an improvement in the symptoms (67/122, 54.9%).

**Table 4:** The reasons for leaving the emergency department.

Reasons	n (%)
Symptom improved	67 (55%)
Long wait	19 (16%)
Sufficient information at triage	13 (11%)
Other priorities	7 (6%)
Emergency department crowding	5 (4%)
others	17 (14%)

## Discussion

The present study, the first to investigate LWBS in a Japanese ED, found the incidence of LWBS to be 0.15%. This LWBS rate was lower than that reported in previous studies, ranging from 0.5-16.6% in pediatric emergency departments in children's hospitals [4-15]. Associated factors in this study were similar to those in previous reports [5,15,17,19,23], suggesting that they may be universal. For example, Lucas J et al. proposed the impact of wait time on the occurrence of LWBS, and Tropea J et al. described the association between triage category and LWBS. Despite this similarity, the LWBS rate varied widely, suggesting that the strength of each factor varied. The factors identified in the present study were compared with those in previous reports.

Acuity level at triage

Goldman RD et al. and other studies suggested that a low acuity triage category is more likely to increase the LWBS rate [5,8,10]. In the present study, level 5 was not shown to be significant, but as in previous reports, the association between low acuity and LWBS was strong. Spontaneous recovery during long waits may be a contributing factor. These findings suggest that acuity at triage is universally related to LWBS.

Day of the week and time of visit

The present study found that LWBS was more likely to occur on weekends/holidays and during the evening hours when the total number of patients was higher than at other times. However, previous reports did not match the highest, total number of patients with the time of day when most LWBS cases occurred [10,15], suggesting that the total number of patients per period was only one factor leading to LWBS. Crowding in emergency departments can fluctuate rapidly over very short periods owing to a variety of reasons related to supply and demand [24]. However, certain time segments may nullify this variability. Therefore, generalizing that congestion leads to LWBS on the basis of the total number of patients during a specific time period is questionable. Identifying peak hours or time periods with high LWBS rates at each facility and implementing strategies such as increasing staffing levels during these times could be beneficial. Additionally, developing a system to request assistance from available staff during sudden surges in patient volume, such as in disaster situations, could be an effective strategy.

EDLOS and examination wait time

The present study found a strong association between EDLOS and waiting time ORs in LWBS cases. As 44.8-92.7% of LWBS patients in previous studies cited waiting time as a reason for leaving the hospital [5,6,18,25], EDLOS and waiting time appear to be universal and strongly associated. As these factors may be interrelated, it is important to determine which is dominant. Our analysis suggested that waiting time was more strongly associated with LWBS. Waiting time is likely a direct factor, as EDLOS includes time segments that are not applicable to LWBS patients (e.g., time for test). Therefore, waiting time may be a more appropriate index for reducing LWBS than EDLOS. However, while an EDLOS target of less than four hours has been advocated in some countries, there are no recommendations for waiting time targets in the context of crowding [26]. It is more important to seek an appropriate target for waiting time with a view to reducing LWBS, at least in settings where EDLOS of less than four hours is highly achievable.

In the present study, brief crowding may have led to a small number of LWBS in patients with a less urgently acute complaint. This hypothesis is supported by the very low LWBS rate and consistently high EDLOS of less than four hours achievement rate obtained in this study. The potential association between crowding and LWBS suggests that a crowding score may help monitor the LWBS risk [27]. Overcrowding leads to the expectation of prolonged waiting; approximately 49.9% of patients reportedly overestimated waiting times [28]. Therefore, any attempt to address the problem of LWBS may require a strategy to lessen overcrowding, expedite the flow of low-acuity patients facing extended waits, and deliver more accurate waiting time information to patients.

Another important finding of the present study was that there was only one case of LWBS that went unnoticed. In contrast, previous studies have shown that characteristically, a certain number of patients leave the hospital without notification or cannot be contacted after leaving [5,6,12,18,25]. This discrepancy may reflect the cultural and social background of Japan, such as the meticulous and patient nature characteristic of Japanese people. Alternatively, it could potentially underscore the effectiveness of the center's well-established, nurse-led counseling service. The minimal rate of unnoticed discharges in Japan provides some insight into the factors and circumstances influencing the LWBS rate.

This study has several limitations. First, it was retrospective; therefore, the prognosis of patients seeking care elsewhere and potential adverse events related to LWBS might have been under-represented. However, as the study center is the only advanced treatment center in the region, it is likely that any post-LWBS deterioration in the patients would have been referred back to the study center. Therefore, serious outcomes were unlikely in these patients. Second, while the examination times were automatically recorded, pre-populated data and missing examination starting time due to technical issues might have introduced a bias in the estimates of the waiting time. Nevertheless, the large sample size of 81,828 patients after exclusion ensures that the data are reliable. Third, using a single rater to categorize cancellation reasons may introduce rater bias into the analysis. Last, cases of cancellation before triage were not included because they were automatically removed from the system.

## Conclusions

The LWBS rate in the present study was very low compared to those in previous reports. Low acuity, weekend/holiday visits, evening visits, EDLOS of more than one hour, and waiting time of more than one hour were associated with LWBS and were similar to the findings of previous performed in other countries, suggesting that they might be highly generalizable. It is more important to seek an appropriate target for waiting time with a view to reducing LWBS, in settings where EDLOS of less than four hours is highly achievable.
